# Supplementary material for: Metabolic modeling of sex-specific liver tissue suggests mechanism of differences in toxicological responses
Source: PLoS Comput Biol. 2023 Aug 21;19(8):e1010927. doi: 10.1371/journal.pcbi.1010927 (PMC10470949; doi:10.1371/journal.pcbi.1010927)
Supplement: S1 Fig — The results of a TIDEs score comparison of male- and female-sourced non-treated hepatocytes and drug-treated hepatocytes with TIDEs are shown with each row representing a different treatment and each column representing a different subsystem. Each rectangle represents the TIDEs score for a subsystem (column) when treated with a drug (row). Each subsystem was found to be sex-biased in our TIDEs analysis. We find several of these subsystems also have an altered behavior in response to known hepatotoxicants. (DOCX) [file pcbi.1010927.s001.docx]

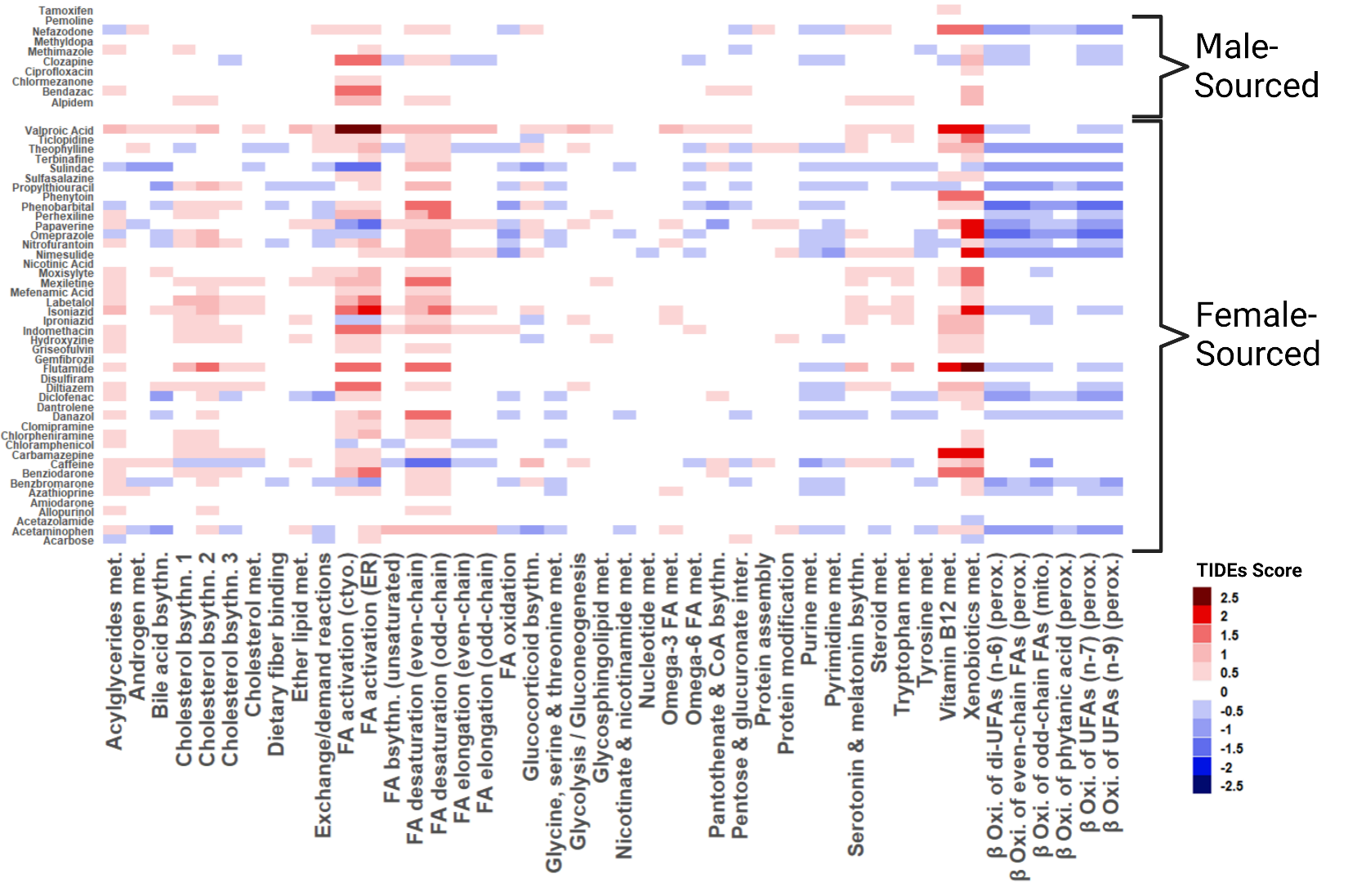


*Supplementary Figure 1:* ***Heatmap with drug-induced TIDEs scores including specific drugs used for each row.*** *The results of a TIDEs score comparison of male- and female-sourced non-treated hepatocytes and drug-treated hepatocytes with TIDEs are shown with each row representing a different treatment and each column representing a different subsystem. Each rectangle represents the TIDEs score for a subsystem (column) when treated with a drug (row). Each subsystem was found to be sex-biased in our TIDEs analysis. We find several of these subsystems also have an altered behavior in response to known hepatotoxicants.*
