# Supplementary material for: Metabolic modeling of sex-specific liver tissue suggests mechanism of differences in toxicological responses
Source: PLoS Comput Biol. 2023 Aug 21;19(8):e1010927. doi: 10.1371/journal.pcbi.1010927 (PMC10470949; doi:10.1371/journal.pcbi.1010927)
Supplement: Attachment — Submitted filename: Review of PCOMPBIOL-D-23-00166.pdf [file pcbi.1010927.s002.pdf]

Review of PCOMPBIOL-D-23-00166
Due 27 Feb 2023

General comments

This study described the differences in metabolism between the male and female liver. Specifically, they used Tasks inferred from Differential Expression (TIDEs) to analyze gene expression differences between the sexes, and found that androgen, ether lipid, glucocorticoid, tryptophan, and xenobiotic metabolism have more activity in the male liver than the female liver. Additionally, sex-specific transcriptomic data showed differences in the import of bile acids and salts, suggesting that the sexually dimorphic behaviour of the liver may be caused by enterohepatic recirculation.

This topic is very interesting and extremely important for toxicity testing. The use of TIDEs and their derivatives to analyse liver transcriptomics is, to my knowledge, novel and will be of interest to the scientific community. The findings are appropriately discussed and linked to the literature. The general workflow of the study is clear, and can be translated to investigate the metabolism of other organs.

I believe that substantial revision will be required for publication in this journal. Below, I provide some suggestions that I think could improve this study.

Major points:

A. The results of the first paragraph and Figure 1 do not seem to support the statement “Women experience liver-related adverse events more frequently than men”.
A robust analysis of liver-related adverse events requires a multivariate model that accounts for age, drug type (and whether it’s used more by women) and socioeconomic factors, etc. A univariate analysis is misleading and substantially lowers the quality of this work. Given the strong premise outlined in the introduction (lines 61-64), I would recommend considering removing this section.

B. The gene expression dataset GSE130991 contains patients with highly variable characteristics:
https://www.ncbi.nlm.nih.gov/pmc/articles/PMC6545676/
It does not seem that any of this information (which is available) is accounted, based on their repository (https://github.com/ConnorMoore1/Sex-Spec-Hepato/blob/main/R/GSE130991%20DEGs.Rmd). I would recommend accounting for at least age and statin intake.

C. I did not find a clear methods paragraph related to the analysis of hepatocyte expression. I had a look at the GitHub repo, and only found the plotting script:
https://github.com/ConnorMoore1/Sex-Spec-Hepato/blob/main/R/Drug%20Heatmap.Rmd
Please specify how the data was downloaded and processed.

D. To my understanding, the analyses related to Figure 3 seek to investigate the sex-specific “hepatotoxic side effects of ~~all~~ drugs”. I think to investigate the drug-sex interaction, we would require 4 conditions: drug-treated male

hepatocytes, untreated male hepatocytes, drug-treated female hepatocytes, untreated female hepatocytes. For example, we would expect to see that a gene cluster would be differentially expressed in drug-treated female hepatocytes compared to untreated female hepatocytes, while unchanged in drug-treated male hepatocytes compared to untreated male hepatocytes or vice versa.

What sex were the hepatocytes from? From a quick inspection of the Open TG-GATEs dataset, which I guess is where the raw data from ToxicoDB comes from, it was clear that different compounds were used for different sexes:
https://academic.oup.com/nar/article/43/D1/D921/2439524#supplementary-data/

I, therefore, believe that this dataset is inappropriate to investigate sex-related differences because: 1. It does not have suitable experimental conditions, 2. The sex of the hepatocytes is scrambled in the original data. My suggestion would be to either validate and cross-check these findings using an independent dataset, or to remove this section.

E. The analysis of primary hepatocytes should also be carefully considered. Gene expression changes due to culturing conditions. I am not an expert on culturing primary hepatocytes per se, but a previous study showed that the cellular dynamics and metabolism of primary hepatocytes changed during culture: https://pubmed.ncbi.nlm.nih.gov/34241659/. Please reconsider the use of this dataset and validating some of the results using an orthogonal dataset.
F. Following the above suggestions, the title “Metabolic modeling of sex-specific tissue predicts mechanisms of differences in toxicological responses” is somewhat problematic. 1. The emphasis is on liver, so it should be liver instead of tissue. 2. A subtitle of the results section is “Hepatocyte expression data suggests the metabolic response to hepatotoxic pharmaceuticals does not overlap with sex-biased metabolism”, indicating that the metabolic modeling of sex-specific tissue doesn’t predicts mechanisms of differences in toxicological responses. Please clarify this.

Minor points:

1. Line 129: why were the brain and kidney chosen?
2. Regarding the y-axis in Figure 3, it would be nice to have the full list of drug names, in case anyone would be interested in the effect of a specific drug analysed.
3. Generally, there is no annotation to the scripts. They’re very hard to read. Please consider adding annotations to clarify the steps of analysis. This would allow the readers to better understand and reproduce results.
4. Please discuss the results from Figures 2,4,5 integratively. For examples, can bile acid metabolism be linked to anything in Figure 2C?
5. Please consider including a short description of the datasets used in each section.
