# Supplementary material for: Metabolic modeling of sex-specific liver tissue suggests mechanism of differences in toxicological responses
Source: PLoS Comput Biol. 2023 Aug 21;19(8):e1010927. doi: 10.1371/journal.pcbi.1010927 (PMC10470949; doi:10.1371/journal.pcbi.1010927)
Supplement: Attachment — Submitted filename: Response to Reviewers.pdf [file pcbi.1010927.s003.pdf]

We thank the reviewers for their comments and insight. Their contributions have greatly improved the quality of the manuscript. The original text of the reviews are shown in **black**, and our response to each comment is shown in **blue**.

—----------------------------

Reviewer #1: In this study, the authors utilize GEM to analyze sex-dependent differences between female and male with regard to metabolic alteration in toxicological responses mostly in liver, but also in kidney and brain. The authors identified several metabolic pathways (subsystems) that are dysregulated in liver between male and female. In general, the manuscript is well written; methods and results are clearly presented and sufficiently discussed. This study provides insight into how sex affects drug response in the treatment of liver diseases, which is an important step toward precision medicine. I have number of minor comments.

We thank the reviewer for the positive evaluation of the manuscript and the helpful comments and critiques.

1. Line 149 – 150. It is unclear if the author is saying that the two metabolic pathways were upregulated in males in both liver and kidney, or either in liver or in kidney (when using the term "between the liver and kidney").

We have reworded this section to clarify that the pathways are upregulated in both tissue types.

2. Line 145. The authors reported that 11 metabolic subsystems were dysregulated in liver. However, in the following text the authors only mentioned two (acylglyceride and steroid) and the other eight metabolic subsystems in Figure 2C.

We have edited the manuscript to fix this error.

3. Line 154 – 171. Most of the content seems like discussions rather than results. I suggest the structuring of the results and discussions carefully.

We have moved this content from Results to Discussion.

4. Line 175 – 176. As this is the first sentence, it is better to indicate what cells/materials were used in addition to the words “treatment and non-treatment conditions”.

We have added the fact that these cells are primary human hepatocytes.

5. Line 155. I guess here it should be Figure 2C not Figure 3A.

We have edited the manuscript to fix this error.

Reviewer #2:

General comments

This study described the differences in metabolism between the male and female liver. Specifically, they used Tasks inferred from Differential Expression (TIDEs) to analyze gene expression differences between the sexes, and found that androgen, ether lipid, glucocorticoid, tryptophan, and xenobiotic metabolism have more activity in the male liver than the female liver. Additionally, sex-specific transcriptomic data showed differences in the import of bile acids and salts, suggesting that the sexually dimorphic behaviour of the liver may be caused by enterohepatic recirculation.

This topic is very interesting and extremely important for toxicity testing. The use of TIDEs and their derivatives to analyse liver transcriptomics is, to my knowledge, novel and will be of interest to the scientific community. The findings are appropriately discussed and linked to the literature. The general workflow of the study is clear, and can be translated to investigate the metabolism of other organs.

I believe that substantial revision will be required for publication in this journal. Below, I provide some suggestions that I think could improve this study.

We appreciate the positive evaluation of the reviewer and the helpful comments below. We note that with the additional analysis we focused more of the paper’s discussion on differences between male and female models in the pentose phosphate pathway given the role of oxidative stress in drug response. We still report the role of xenobiotic and bile acid-related metabolism that differ between the male and female liver gene expression data but place the greater focus on oxidative stress given its relevance.

Major points:

A. The results of the first paragraph and Figure 1 do not seem to support the statement “Women experience liver-related adverse events more frequently than men”.

A robust analysis of liver-related adverse events requires a multivariate model that accounts for age, drug type (and whether it’s used more by women) and socioeconomic factors, etc. A univariate analysis is misleading and substantially lowers the quality of this work. Given the strong premise outlined in the introduction (lines 61-64), I would recommend considering removing this section.

We agree that a multivariate model that accounts for many of these confounding variables would be powerful for addressing many such questions. Unfortunately, much of that required data is simply not available. We have edited the text to reflect this important concern and added corresponding citations.

B. The gene expression dataset GSE130991 contains patients with highly variable characteristics:

https://www.ncbi.nlm.nih.gov/pmc/articles/PMC6545676/

It does not seem that any of this information (which is available) is accounted, based on their repository

(https://github.com/ConnorMoore1/Sex-Spec-Hepato/blob/main/R/GSE13099 1%20DEGs.Rmd). I would recommend accounting for at least age and statin intake.

We have better controlled our data to those patients between the ages of 18 and 50 who are not using statins and report the resulting analysis in the manuscript.

C. I did not find a clear methods paragraph related to the analysis of hepatocyte expression. I had a look at the GitHub repo, and only found the plotting script: https://github.com/ConnorMoore1/Sex-Spec-Hepato/blob/main/R/Drug%20He atmap.Rmd

Please specify how the data was downloaded and processed.

We have added this information to the “Gene expression analysis” section of the methods.

D. To my understanding, the analyses related to Figure 3 seek to investigate the sex-specific “hepatotoxic side effects of all drugs”. I think to investigate the drug-sex interaction, we would require 4 conditions: drug-treated male

hepatocytes, untreated male hepatocytes, drug-treated female hepatocytes, untreated female hepatocytes. For example, we would expect to see that a gene cluster would be differentially expressed in drug-treated female hepatocytes compared to untreated female hepatocytes, while unchanged in drug-treated male hepatocytes compared to untreated male hepatocytes or vice versa.

What sex were the hepatocytes from? From a quick inspection of the Open TG-GATEs dataset, which I guess is where the raw data from ToxicoDB comes from, it was clear that different compounds were used for different sexes:

https://academic.oup.com/nar/article/43/D1/D921/2439524#supplementary-da ta/

I, therefore, believe that this dataset is inappropriate to investigate sex-related differences because: 1. It does not have suitable experimental conditions, 2. The sex of the hepatocytes is scrambled in the original data. My suggestion would be to either validate and cross-check these findings using an independent dataset, or to remove this section.

We agree that the comparisons proposed by this reviewer would facilitate a much stronger analysis. However, we simply don’t have access to the data needed and consequently focus our modeling and analysis on what we can state from what’s available. In this revised manuscript, we have better annotated these data and modified the text to better reflect what conclusions can be made.

E. The analysis of primary hepatocytes should also be carefully considered. Gene expression changes due to culturing conditions. I am not an expert on culturing primary hepatocytes per se, but a previous study showed that the cellular dynamics and metabolism of primary hepatocytes changed during culture: https://pubmed.ncbi.nlm.nih.gov/34241659/. Please reconsider the use of this dataset and validating some of the results using an orthogonal dataset.

In this revised manuscript, we have emphasized the limitation of *in vitro* experiments and the important consideration of culturing conditions. We also include a reference to this paper which emphasizes that with these important caveats, cell culture remains an essential part of hepatic studies.

F. Following the above suggestions, the title “Metabolic modeling of sex-specific tissue predicts mechanisms of differences in toxicological responses” is somewhat problematic. 1. The emphasis is on liver, so it should be liver instead of tissue. 2. A subtitle of the results section is “Hepatocyte expression data suggests the metabolic response to hepatotoxic pharmaceuticals does not overlap with sex-biased metabolism”, indicating that the metabolic modeling of sex-specific tissue doesn’t predicts mechanisms of differences in toxicological responses. Please clarify this.

We have changed our title and the subtitle appropriately to address this concern.

Minor points:

1. Line 129: why were the brain and kidney chosen?

We use these tissues as representative examples of sexually dimorphic and sexually monomorphic tissue types. We clarified this reasoning in the manuscript.

2. Regarding the y-axis in Figure 3, it would be nice to have the full list of drug names, in case anyone would be interested in the effect of a specific drug analysed.

We have added a supplementary figure that includes these details.

3. Generally, there is no annotation to the scripts. They’re very hard to read. Please consider adding annotations to clarify the steps of analysis. This would allow the readers to better understand and reproduce results.

We have updated the scripts to be more clearly accessible.

4. Please discuss the results from Figures 2,4,5 integratively. For examples, can bile acid metabolism be linked to anything in Figure 2C?

We have added observations from an integrated consideration of the data in the figures to our Discussion section.

5. Please consider including a short description of the datasets used in each section.

We added a short description about the datasets in our methods section.

Reviewer #3: Summary

The authors Integrate gene expression data to contextualize the previously published Human1 human cell metabolic network model to characterize the impact of transcriptional changes of metabolic genes in the context of sex differences and drug treatment.

They used Tasks Inferred from Differential Expression (TIDEs) to discover that androgen, ether lipid, glucocorticoid, tryptophan, and xenobiotic metabolism have more activity in the male liver, and serotonin, melatonin, pentose, glucuronate, and vitamin A metabolism have more activity in the female liver. On drug treatment in hepatocytes the largest differences berween the sexes are in subsystems related to lipid metabolism. sex-specific transcriptomic data was used to create individual and averaged male and female liver models. The results indicate that the sexually dimorphic behavior of the liver may be caused by differences in enterohepatic recirculation. The authors suggest an investigation into sex-specific microbiome composition for further understanding. Moreover, the authors point out the male-bias in clinical testing of drugs that has unfortunately led to a disproportionate number of hepatotoxic events in women and reiterate the lack of focus on the systematic interactions of these differences.

We thank the reviewer for their positive evaluation of the manuscript and the helpful comments below.

Comments:

There are no major issues with this study as detailed below. Based on the technical and scientific soundness of the study, I recommend publication.

(1) The question the authors are trying to answer is very relevant and the need of the hour

We appreciate the reviewer recognizing the need for this investigation.

(2) Overall, the findings reported here can help shape sex-specific research to represent the entire human population and potentially guide future drug design and toxicological testing.

We thank the reviewer for their optimistic vision of the future of this work.

(3) There are some minor typographical errors and I recommend correcting them before publication

We have fixed several minor errors in the manuscript.
